# Expression of the Receptor Tyrosine Kinase EphB2 on Dendritic Cells Is Modulated by Toll-Like Receptor Ligation but Is Not Required for T Cell Activation

**DOI:** 10.1371/journal.pone.0138835

**Published:** 2015-09-25

**Authors:** Patrice N. Mimche, Lauren M. Brady, Shirley Keeton, David S. J. Fenne, Thayer P. King, Kendra M. Quicke, Lauren E. Hudson, Tracey J. Lamb

**Affiliations:** 1 Division of Infectious Diseases, Department of Pediatric, Emory University School of Medicine, Emory University, Atlanta, Georgia, United States of America; 2 Institute for Cardiovascular and Metabolic Research, School of Biological Sciences, University of Reading, Reading, Berkshire, United Kingdom; Rutgers University, UNITED STATES

## Abstract

The Eph receptor tyrosine kinases interact with their ephrin ligands on adjacent cells to facilitate contact-dependent cell communication. Ephrin B ligands are expressed on T cells and have been suggested to act as co-stimulatory molecules during T cell activation. There are no detailed reports of the expression and modulation of EphB receptors on dendritic cells, the main antigen presenting cells that interact with T cells. Here we show that mouse splenic dendritic cells (DC) and bone-marrow derived DCs (BMDC) express EphB2, a member of the EphB family. EphB2 expression is modulated by ligation of TLR4 and TLR9 and also by interaction with ephrin B ligands. Co-localization of EphB2 with MHC-II is also consistent with a potential role in T cell activation. However, BMDCs derived from EphB2 deficient mice were able to present antigen in the context of MHC-II and produce T cell activating cytokines to the same extent as intact DCs. Collectively our data suggest that EphB2 may contribute to DC responses, but that EphB2 is not required for T cell activation. This result may have arisen because DCs express other members of the EphB receptor family, EphB3, EphB4 and EphB6, all of which can interact with ephrin B ligands, or because EphB2 may be playing a role in another aspect of DC biology such as migration.

## Introduction

Erythropoietin-producing hepatocellular (Eph) receptors were first discovered in 1987 [1] and are the largest family of receptor tyrosine kinases. They have been split into two groups based on sequence conservation—the A and B families—and these receptors bind Ephrin A and B ligands respectively. There are now several reports describing the expression of Eph receptors and Ephrin ligands of both the A family [2–7] and the B family [3, 8–14] on the surface of immune cells, yet the function of this family of molecules in immune responses is currently unclear.

T cells have been reported to express both Ephrin ligands and Eph receptors on their surface. Ephrin B ligands have been shown to play a role in the development of T cells in the thymus [9, 13], possibly by stabilizing the expression of the IL-7Rα at the surface of the developing T cells [15]. T cells require co-stimulatory signals in addition to ligation of the T cell receptor (TCR) to become activated. Ephrins B1 [12], B2 [10] and B3 [11] have been suggested to function in co-stimulation of T cells. If Ephrin B ligands are co-stimulatory molecules, they should ligate with EphB receptors expressed on the surface of dendritic cells (DC), the main antigen presenting cells that activate T cells. The ligation of Ephrin ligands with Eph receptors results in bi-directional signaling [16] that may act to potentiate signals in the T cell as well as the activating DCs. Indeed, Ephrin B expression on T cells has been shown to be required for optimal IL-6 receptor ligation-induced STAT3 phosphorylation in T cells [9] demonstrating that ligation with EphB receptors has the potential to induce signaling pathways that may influence the response of T cells to cytokine stimulation.

Although there have been reports of Eph receptor expression on some human DC subsets with EphB3 expressed by Langerhans cells, EphA2 and EphA7 expressed by interstitial DCs and EphB1 on plasmacytoid DCs [3, 17, 18] there have been no reports investigating how these molecules are regulated on DCs or whether they alter T cell responses upon activation, as might be expected from co-stimulatory molecules. Given that monocytes/macrophages express EphB2 [19, 20] and Ephrin B ligands are present on T cells, we hypothesized that EphB2 would be expressed on DCs and its deficiency on this immune cell type might result in altered T cell expansion and activation. The data reported here show that splenic DCs and bone marrow-derived DCs (BMDCs) express EphB2 on their surface and that this receptor is up-regulated by both Toll-like receptor (TLR) ligation and ligation with Ephrin B ligands. We show that EphB2 co-localizes with major histocompatibility complex (MHC)-II on BMDCs but EphB2-/- deficiency on BMDCs does not impair T cell activation. Taken together, these results reveal that EphB2 is expressed by DCs yet is not required to activate T cells. This may be due to the expression of other EphB receptors on BMDCs including EphB3, EphB4 and EphB6 that may functionally compensate for the absence of EphB2. However this does not exclude another role for EphB2 on DCs, such as in facilitating migration to the draining lymph nodes from areas of antigenic challenge.

## Materials and Methods

### Ethics statement

Animals were housed in sterile cages and kept under conventional conditions. All experiments were approved by the Emory University Institute for Animal Care and Use Committee and conducted in accordance with approved guidelines.

### Mice

Female C57BL/6 and T cell receptor OT-II transgenic mice [21] were maintained by the Division of Animal Resources on the Emory campus or purchased from The Jackson Laboratory (Bar Harbor, ME, USA). EphB2-/- mice on a C57BL/6 background [22] were a kind gift from Jonathan Gibbins (University of Reading, UK) and were re-derived and bred under a heterozygote breeding system to allow comparison of homozygote EphB2-/- mice with wild type littermate control mice. Experiments were conducted at the Emory University Department of Pediatrics Animal Facility on female mice aged 6–12 weeks that were fed diet 5001 (LabDiet, MO, USA) and given food and water *ad libitium*. Mice were euthanized by CO_2_ inhalation.

### Cell lines

The interleukin (IL)-2 sensitive cell line, HT-2 (American Type Culture Collection (ATCC), Manassas, VA) was maintained in RPMI-1640 supplemented with 10% heat inactivated fetal calf serum (FCS) (PAA Laboratories), penicillin (100U/ml) streptomycin (100μg/ml), L-glutamine (2mM), 2-mercaptoethanol (50μM), sodium pyruvate (1mM), 4-(2-hydroxyethyl)-1-piperazineethanesulfonic acid (HEPES) (10mM) (all Life Technologies / Invitrogen) plus human recombinant IL-2 (150U/ml) (PeproTech). The T cell hybridoma MF2.2D9 with T cell receptors reactive to chicken ovalbumin (OVA)_257-278_ (a kind gift from Kenneth Rock, University of Masachusetts [23]) was cultured in DMEM supplemented with 10% FCS, penicillin (100U/ml) streptomycin (100μg/ml), L-glutamine (2mM), 2-mercaptoethanol (50μM), sodium pyruvate (1mM), HEPES (10mM).

### Mouse DC Preparations

For the isolation of BMDCs, mice (n = 2) were sacrificed by CO_2_ inhalation and bone marrow was removed from the femur and tibia by flushing the shaft with Iscoves’ Modified Eagles Medium (IMDM) (Life Technologies / Invitrogen). Red blood cells (RBC) were lysed using RBC lysis buffer (eBioscience), and the remaining cells were plated in a 6-well tissue culture plate at a density of 10^6^ cells/ml per well in IMDM supplemented with 10% heat inactivated FCS (PAA laboratories), penicillin (100U/ml) streptomycin (100μg/ml), L-glutamine (2mM), 2-mercaptoethanol (50μM), sodium pyruvate (1mM), HEPES (10mM) (all Life Technologies / Invitrogen) with additional recombinant mouse granulocyte macrophage colony stimulating factor (GM-CSF) (Miltenyi Biotech) at a concentration of 40ng/ml. The culture medium was replenished at days 3 and 5, and the loosely adherent BMDCs were harvested at day 7 of culture and used for further experiments. To prepare splenic DCs, spleens from 10 mice were incubated in Liberase TL (Roche) for 30 minutes at 37°C at a concentration of 0.4mg/ml and splenocytes aseptically isolated before lysing RBCs with RBC lysis solution (eBioscience). CD11c+ cells were purified using Miltenyi MACS separation beads (Miltenyi Biotech) according to the manufacturer’s instructions.

### Human DC preparations

Human blood was used for isolation of human DCs and obtained through a blood donation center (LifeSouth; http://www.lifesouth.org/drives/). Human monocyte derived dendritic cells (MoDC) (a kind gift from Jan Mead, Emory University, USA) were prepared according to the protocol by Bedi and Mead [24]. Briefly, peripheral blood mononuclear cells (PBMCs) were isolated from buffy coats of heparinized blood from healthy volunteers (LifeSouth, US) and CD14+ cells isolated and cultured in the presence of recombinant human GM-CSF and IL-4 for 5 days until cells up-regulated CD11c and CD11b.

### DC Stimulation

BMDCs (1x 10^6^) were cultured with lipopolysaccharide (LPS) from *Salmonella minnesota* (Alexis) at a concentration of 1μg/ml or CpG1668 (TCCATGACGTTCCTGATGCT with phosphorothioate linkages; Life Technologies / Invitrogen) at a concentration of 1μM for different amounts of time. Samples were plated in duplicate. In some experiments BMDCs were cultured on plate-bound recombinant mouse Ephrin B1-Fc or Ephrin B2-Fc chimeric proteins (R and D systems) at a concentration of 5 μg/ml. Stimulation was carried out in the presence or absence of 20ng/ml of recombinant mouse interferon-γ (IFN-γ) (eBioscience).

### Flow Cytometry

Cells were incubated with Fc block (clone 2.4G2) for 20 minutes on ice before surface staining with fluorescently labelled CD11c (clone N418), MHC-II (clone M5/114.15.2), CD4 (GK15), CD8 (clone 53–6.7), Vα2 TCR chain (clone B20.1) antibodies (all from eBioscience). For detection of EphB receptors and Ephrin molecules on splenic CD11c^hi^ DC, cells were incubated with recombinant mouse EphB2-Fc or mouse Ephrin B2-Fc chimeric proteins (R and D systems) at a concentration of 200ng/ml for 30 minutes on ice. Bound molecules were detected by incubation with a secondary biotinylated anti-human IgG Fc antibody (eBioscience) for 20 minutes followed by 15 minutes of incubation with APC-conjugated strepatavidin (eBioscience). T cell cytokines were detected by intracellular cytokine staining after fixation of cells in 2% paraformaldehyde solution and permeabilization using 0.5% saponin solution (Sigma).

### Confocal Microscopy Analysis

BMDCs (10^5^ cells) were cytospun onto glass slides and fixed with 2% paraformaldehyde. Slides were stained with the following primary antibodies or isotype controls: anti-mouse EphB1 polyclonal antibody at a 1:500 dilution (Pierce), anti-mouse EphB2 (clone 512001 or clone 512013 at 2μg/ml; R&D systems), anti-mouse EphB3 monoclonal antibody (clone 521002; R&D systems), anti-mouse EphB4 monoclonal antibody (clone 117808; R&D systems), anti-mouse EphB6 monoclonal antibody (clone 5D8; Novus Biological) or MHC-II-FITC (clone M5/114.15.2; eBioscience), using standard methodology. Slides were then stained with secondary rat or rabbit antibodies labelled with NorthernLights 493^®^ or NorthernLights 577^®^ (R&D Systems) at a dilution of 1:1000 and nuclei counterstained with mounting medium containing DAPI (VectorShield). Images were captured using a Carl Zeiss confocal microscope and analyzed using Image J software.

### Quantitative Real-Time PCR (qRT-PCR)

Cells were homogenized in RNA Stat60^®^ (Tel-Test Inc., TX, USA) and total RNA extracted using standard phenol-chloroform protocols followed by DNase treatment of RNA extracted using Nucleospin RNA-II purification kit (Nachery-Nagel). A total of 100ng of RNA per sample was converted into cDNA using Superscript II (Life Technologies) at 42°C for 50min, 70°C 15min, in the presence of 5μM oligo (dT)_16-18_, 5mM Dithiothreitol (DTT), 0.5mM dNTPs (all Life Technologies), 8U RNAsin (Promega), 50mM Tris-HCl pH8.3, 75mM KCl and 3mM MgCl_2_. The cDNA was treated with 2.5U RNAse H (Affymetrix) at 37°C for 20min to remove any remaining RNA residues. Real-time qPCR reactions were performed using Quantitect SYBR Green PCR reagent (Qiagen). PCR amplification was performed with 5μl cDNA sample (diluted 1:10), 2μM of each primer and 7μl of QPCR SYBR green mix. Plates were run using an Applied BioSystems FAST 7000 Sequence detection system (ABI Prism FAST 7000). Primer sequences are shown in supporting information S1 Table. Transcripts were normalized to two different housekeeping genes (Ubiquitin and β-actin) and expression levels calculated using the 2^-ΔΔCt^ method [25].

### Western Blot

BMDCs lysates were prepared with radio-immunoprecipitation (RIPA) buffer containing 1x EDTA/proteinase-phosphatase inhibitor cocktail (Pierce). The lysate supernatant was stored at -80°C until used for immunoblotting. Protein extracts were separated by SDS-PAGE electrophoresis and blotted onto nitrocellulose membrane. Blots were blocked in 5% fat free milk in 1x TBS-Tween 20 for 1 hour and then incubated overnight with an HRP-tagged mouse anti-phospho-Tyrosine-100 antibody at a 1:500 dilution. Anti-mouse β-actin (clone AC-15; dilution 1:1000; Pierce) was used as a loading control. Blots were then stained for 1 hour with rat HRP-conjugated secondary anti-IgG (R and D Systems) at a dilution of 1:2000. Finally, blots were developed using ECL substrate as per the manufacturer’s instructions (Pierce) and bands quantified using densitometry measurements on Image J software.

### T cell Activation Assay

1 x 10^5^ BMDCs were incubated with 5 x 10^5^ OT-II T cells containing T cell receptors reactive to OVA_323-339_ and 1mg/ml of OVA (Sigma). T cells were purified from single cell splenocyte preparations from 5 naïve OT-II mice using CD4+ Miltenyi MACs beads according to the manufacturer’s instructions or sorted into CD4+Vα2TCR+ cells on a FACS Aria cell sorter (Becton Dickinson). After 5 days, cultured cells were incubated on plate bound anti-CD3 (clone17A2; eBioscience) in the presence of 2μg/ml of anti-CD28 (clone 37.51; eBioscience) and 10μg/ml of brefeldin A (Sigma) for 6 hours before undergoing surface and intracellular staining for analysis by flow cytometry.

### Antigen Presentation Assay

BMDCs were incubated in varying numbers with 2 x 10^4^ MF2.2D9 and either 1 mg/ml of OVA protein or 1μM endotoxin-free OVA_257-278_ peptide (SIINFEKLTEWTSSNVMEERKI) (Biosynthesis) for 18 hours in triplicate. The supernatants were tested for IL-2 secretion using an IL-2 sensitive cell line (HT-2 cell) and proliferation was evaluated using an ATP-based luminescence cell proliferation and viability assay following the manufacturer’s instruction (Cell Titer-Glo^®^ Luminescent, Promega). Luminescence was read on a microplate scintillation and luminescence counter (TopCount NXT, PerkinElmer).

### Luminex

Cytokine analysis was performed using Luminex reagents from Life Technologies / Invitrogen according to the manufacturer’s instructions and a Luminex® 100/200 Analyzer.

### Statistical analysis

Each experiment was completed at least three times, each with an independent biological preparation of cells. Statistical analysis was performed using the Kruskal Wallis one-way analysis of variance and Dunn’s multiple comparison post-test in Graph Pad Prism software. A P value <0.05 was considered statistically significant.

## Results

### Mouse DCs express EphB2 receptor

**Fig 1:**
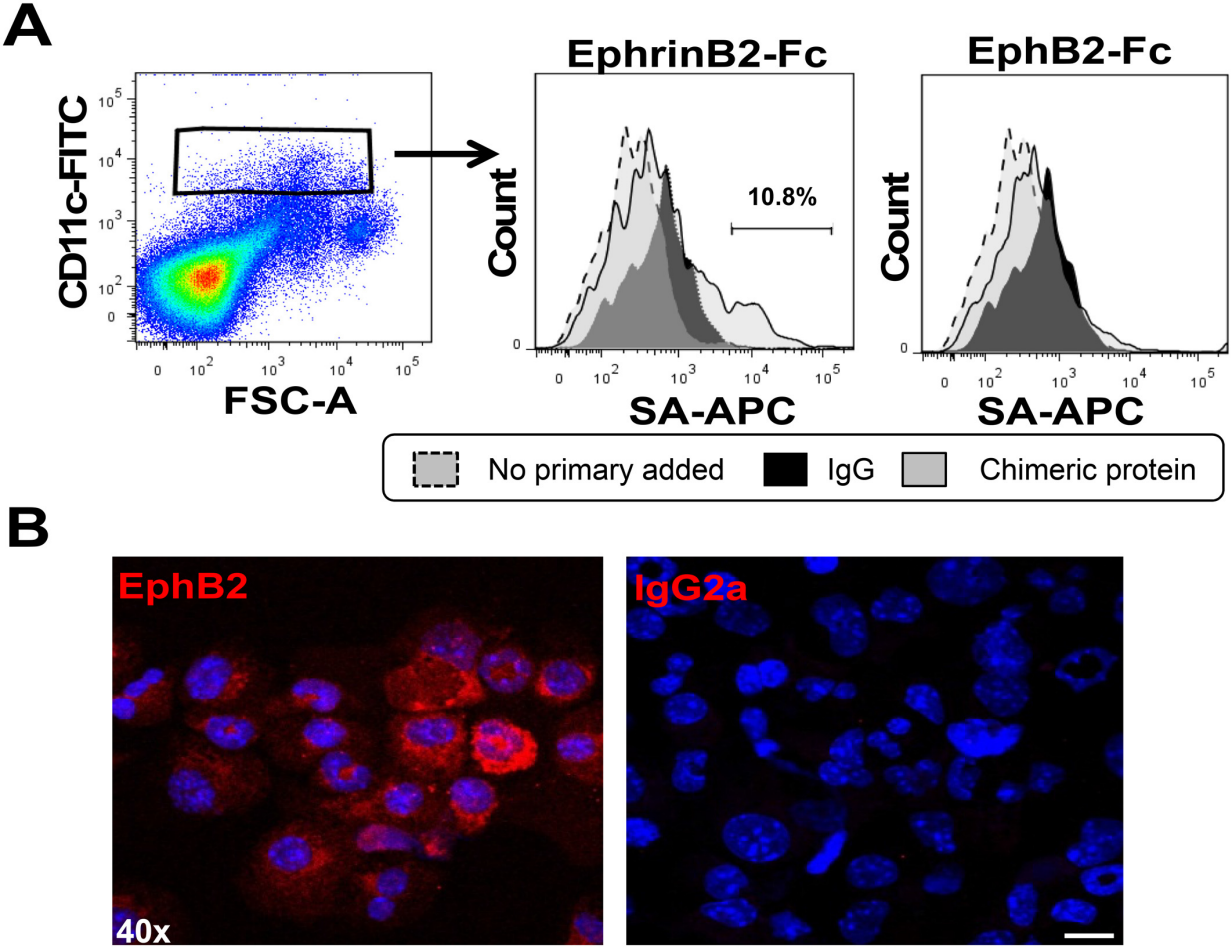
Splenic CD11c^hi^ and bone marrow-derived DCs express EphB2. (A) Eph receptor expression on the surface of naïve splenic CD11c^hi^ DCs was detected by incubation with Ephrin-B2-Fc chimeric protein and binding was detected using a biotinylated anti-Fc antibody and streptavidin-APC (SA-APC) using flow cytometry. (B) The expression of EphB2 on BMDCs incubated with anti-EphB2 antibody or isotype control (IgG2a) detected by immunofluorescence. Magnification 40x; Scale bar, 10μm.

To assess whether mouse DCs express EphB2 receptors, we incubated naïve C57BL/6 mouse splenocytes with recombinant mouse Fc-Ephrin B2, the main ligand for EphB2 receptor. The binding of Ephrin B2 ligand onto expressed EphB receptors was detected by flow cytometry and a subset of CD11c^hi^ DCs bound recombinant EphrinB2-Fc chimeric ligand over and above binding of anti-human IgG alone (used as a negative control) (Fig 1A). This indicates that mouse splenic DCs express EphB receptors. We did not detect any binding of EphB2-Fc chimeric ligand over and above the binding of anti-human IgG alone suggesting that splenic CD11c^hi^ DCs do not express Ephrin B ligands on their surface (Fig 1A). Since there is redundancy in the binding of Eph receptors to ephrin ligand, we assessed whether DC specifically express EphB2. Immunohistochemistry was performed on BMDCs with anti-mouse EphB2 antibody. Labelled secondary antibody only bound to DCs incubated with anti-EphB2 antibody and not those incubated with an isotype control antibody demonstrating a baseline expression of this molecule on unstimulated BMDCs (Fig 1B).

### EphB2 is upregulated on BMDCs in response to TLR ligation

**Fig 2:**
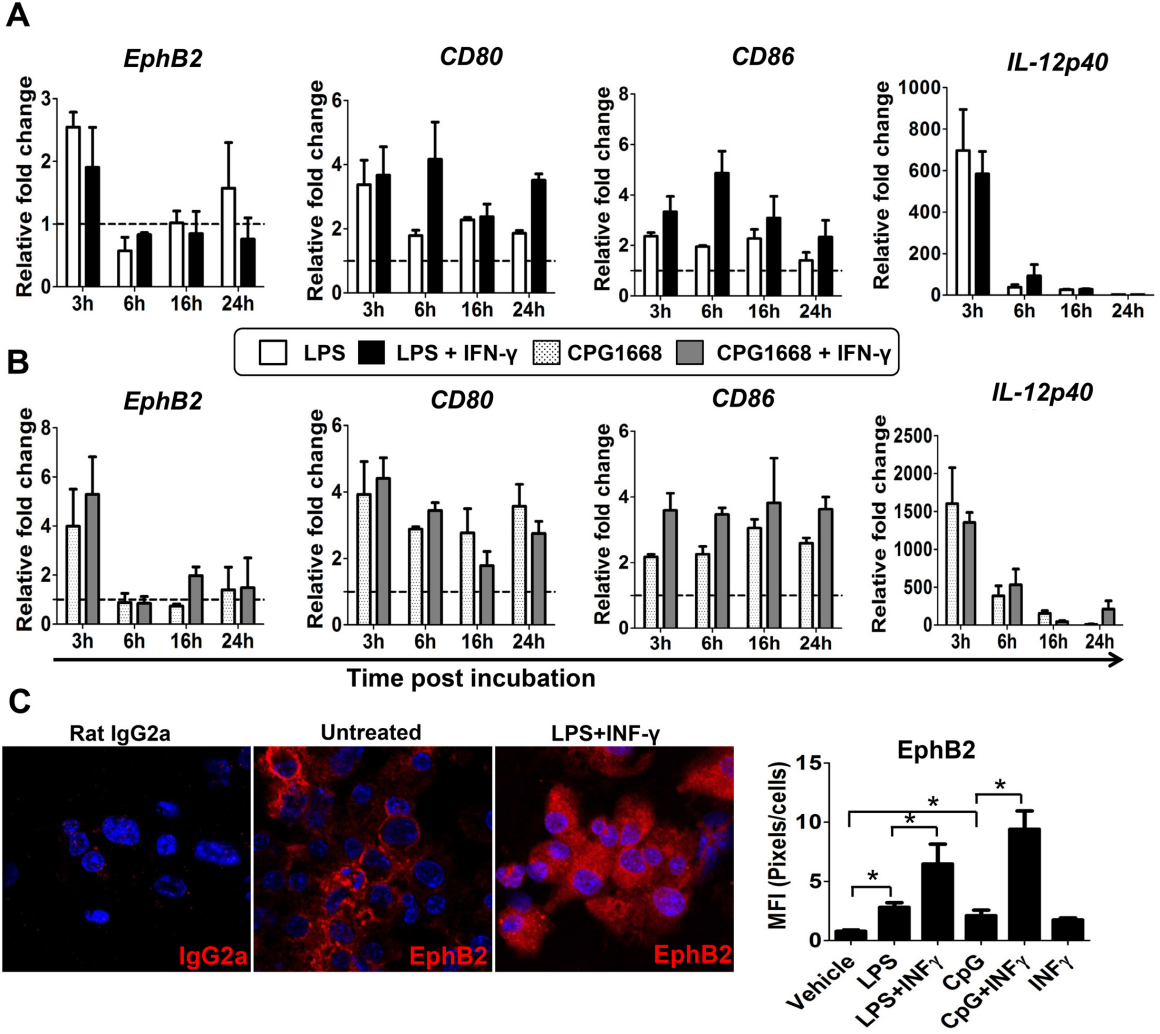
EphB2 expression on BMDCs can be modulated by ligation with Toll-like receptor ligands. (A) BMDC were incubated with a Toll Like receptor (TLR)-4 agonist (lipopolysaccharide (LPS) 1μg/ml) and (B) a TLR-9 agonist (CpG1668 1μM) with or without recombinant mouse interferon (IFN)-γ at a concentration of 20ng/ml. *EphB2* mRNA was quantified by qPCR at different time points post-stimulation. The modulation of transcription for known co-stimulatory molecules CD80 and CD86 as well as the cytokine interleukin (IL)-12p40 in response to TLR stimulation in the same experiments are shown for comparison. (C) The change in EphB2 protein expression at 22 hours post-incubation with LPS and IFN-γ is shown and the mean fluorescence quantified for different conditions. All graphs represent the median value of pooled data across 3 independent BMDC preparations ±SD and data analyzed using One-way ANOVA– Kruskal Wallis test and Dunn’s multiple comparisons post-test. *P<0.05. MFI = Mean Fluorescence Intensity.

To address whether baseline EphB2 expression could be modulated on activated DCs, BMDCs were incubated with bacterial TLR ligands LPS and CpG in the presence or absence of IFN-γ for different amounts of time. The transcription of *EphB2* in response to these stimuli was monitored by qPCR. Transient upregulation of *EphB2* mRNA occurred 3 hours after incubation with LPS or CpG (Fig 2A and 2B) and this was not significantly enhanced by additional stimulation with recombinant IFN-γ. When BMDCs were stimulated with TLR agonists with or without IFN-γ, upregulation of *EphB2* mRNA was between 2.5 and 6 fold increase compared to unstimulated BMDCs at the same time point. Interestingly the magnitude of the change in *EphB2* transcription was in the same range as that for the known co-stimulatory molecules *CD80* and *CD86* (Fig 2A and 2B). The stimulation with LPS and CpG was sufficiently adequate to upregulate *IL-12p40* mRNA by ~700 fold (Fig 2A) and ~1500 fold (Fig 2B) respectively demonstrating that the stimulation by LPS and CpG was successful. There was no significant increase in *EphrinB1*, *B2* and *B3* mRNA expression—in fact transcription of these molecules appears to be downregulated at the same time point (S1 Fig). Altogether these data suggest that treatment of BMDCs with TLR agonists LPS and CpG with or without IFN-γ upregulates transcription of *EphB2* but not ephrin B ligands.

To verify that these changes in transcription of *EphB2* resulted in an increase in the levels of EphB2 protein on the cell surface, immunofluorescence of BMDCs at 22 hours post-stimulation was carried out and quantified according to the fluorescent intensity induced by binding of anti-EphB2 antibody. There was a significant increase in EphB2 protein expression in response to stimulation with both LPS and CpG that was further enhanced in the presence of recombinant IFN-γ (Fig 2C), indicating that TLR stimulation upregulates the expression of EphB2 protein on mouse BMDCs.

### EphB2 deficiency on BMDCs does not affect pro-inflammatory cytokine production when stimulated with TLR agonists *in vitro*

**Fig 3:**
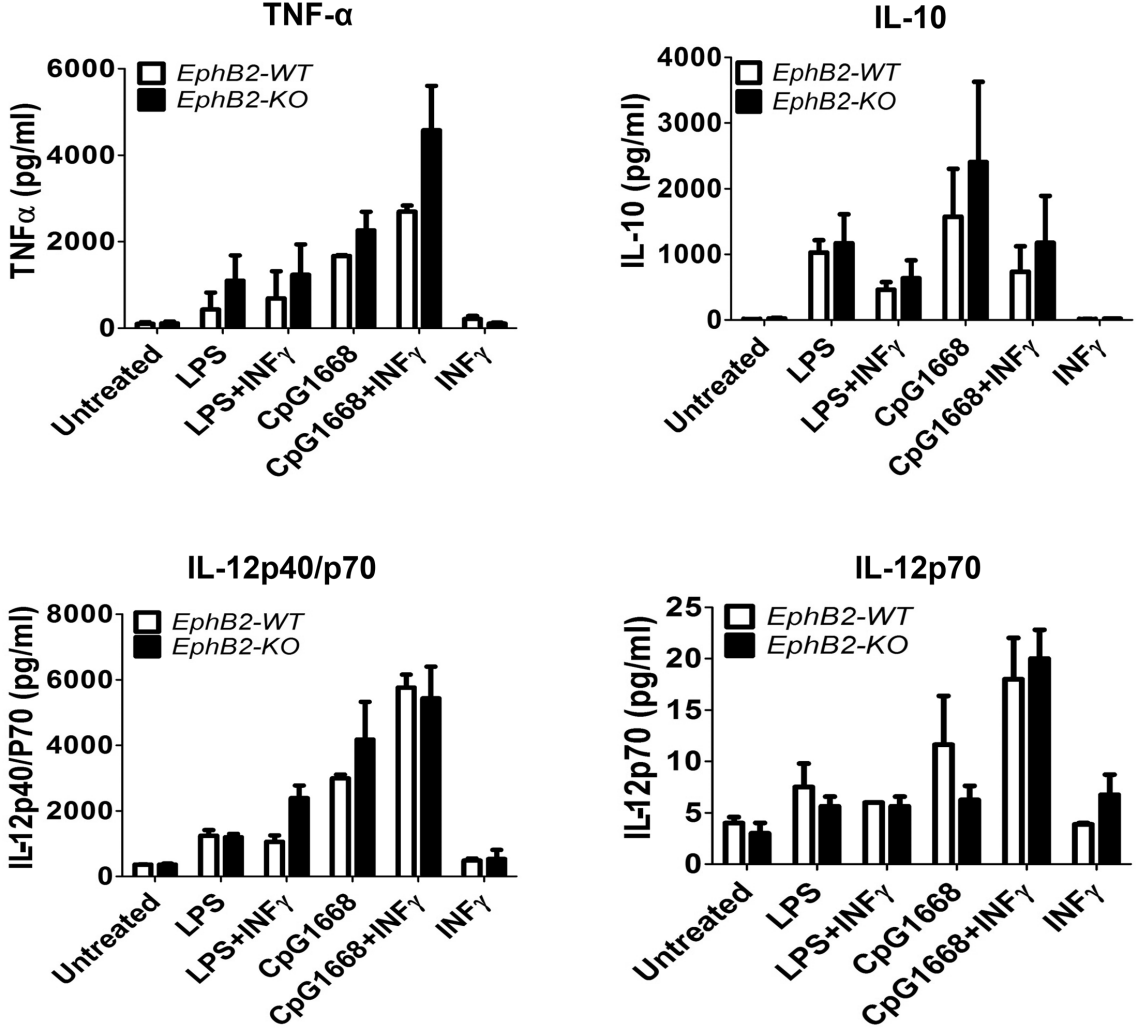
Stimulation of BMDCs from EphB2+/+ and EphB2-/- mice with TLR receptor agonists results in similar levels of secreted cytokine. BMDCs from EphB2+/+ and EphB2-/- mice were incubated with TLR ligands LPS and CpG1668 with and without the addition of recombinant interferon (IFN)-γ for 20 hours. Interleukin (IL)-12p40 (A), IL-12p70 (B), IL-10 (C) and tumor necrosis factor (TNF)-α (D) were measured in the culture supernatant using Luminex. The graphs are representative of 3 individual experiments and bars represent the mean ±SD of 2 replicate wells plated.

Mouse myeloid DC produce inflammatory cytokines when stimulated with TLR agonists [26, 27] and we have shown that EphB2 expression is upregulated by the TLR stimulation. To determine whether EphB2 is involved in the expression of cytokines by DCs activated by TLR ligation, we compared the cytokine secretion profiles of EphB2-/- and EphB2+/+ BMDCs after stimulation with LPS and CpG1668. We found that IL-12p40, IL-12p70, IL-10 and tumor necrosis factor (TNF)-α were produced by LPS and CpG1668 stimulated BMDCs compared to untreated cells (Fig 3). A trend towards increased IL-10 and TNF-α production was observed in EphB2-/- BMDCs compared to EphB2+/+ BMDCs treated with CpG1668 and CpG1668+ IFN-γ respectively (Fig 3). However there were no significant differences in the up-regulation of any of the cytokines measured between EphB2+/+ and EphB2-/- BMDC. Altogether these data show that the absence of EphB2 does not impair cytokine production in BMDCs in response to TLR ligation.

### EphB2 is upregulated on BMDCs in response to ligation with Ephrin B ligands and co-localizes with MHC-II

**Fig 4:**
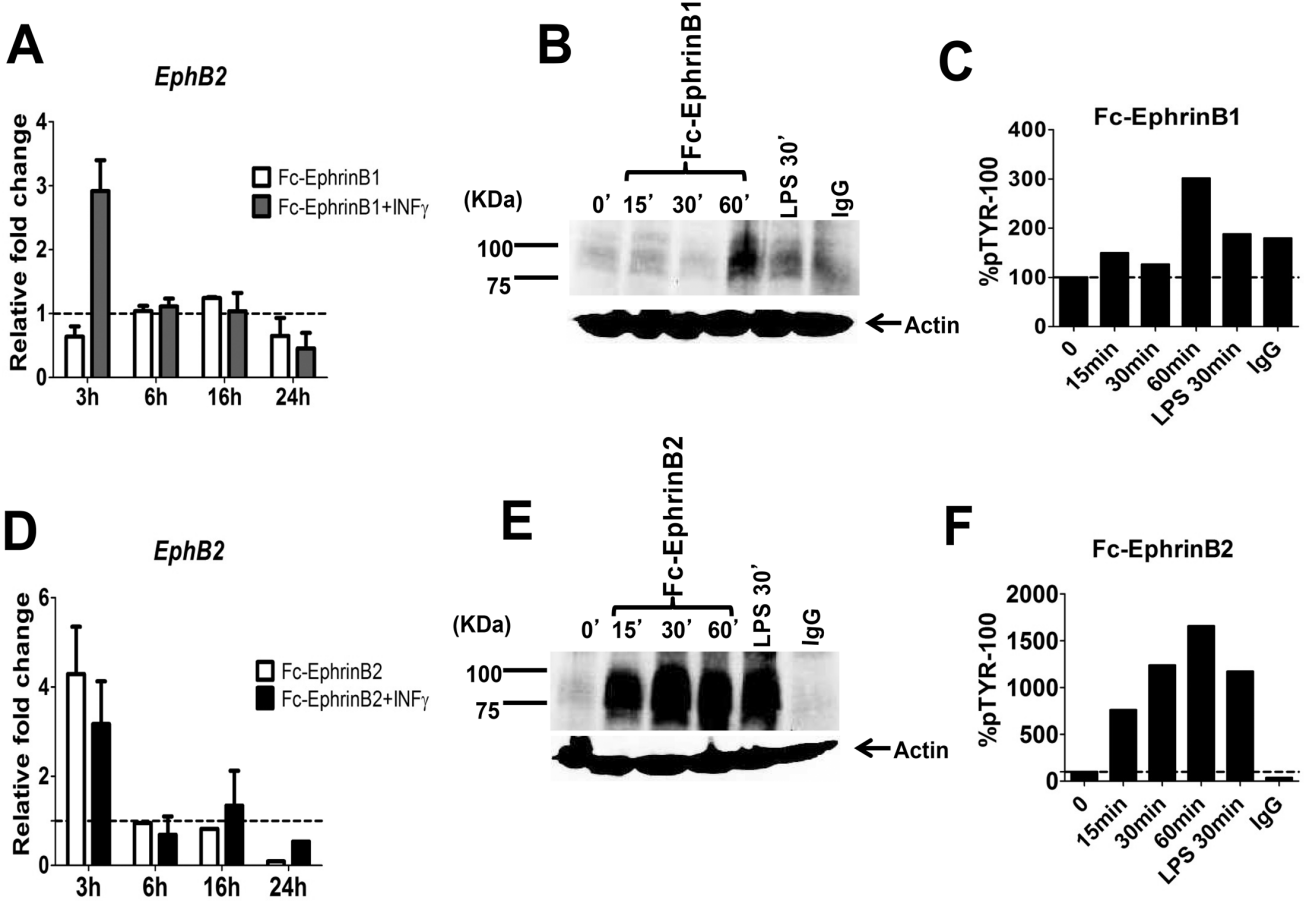
BMDCs stimulated with EphrinB2 upregulate *EphB2* mRNA. Naïve BMDCs were plated onto plate-bound Ephrin-B1-Fc fusion protein (A, B, C) or plate bound Ephrin-B2-Fc fusion protein (D, E, F). The transcription of *EphB2* with and without the addition of recombinant interferon-γ (IFN-γ) was monitored by qPCR over 24 hours of culture (A, D). The efficiency of the Eph receptor ligation was monitored by assessing tyrosine phosphorylation on DCs by western blot (B, E) and the phosphorylation quantified using densitometry (C, F). All graphs represent the median value of pooled data from 3 independent DC preparations ±SD.

During antigenic challenge, CD4+ T cells will only become fully activated upon contact with DCs that present peptide loaded onto MHC-II molecules and display costimulatory molecules that trigger a signalling network within the T cell to induce proliferation and cytokine production. Contact between these molecules also provides feedback signals to the DCs to enhance cytokine production and sustain expression of co-stimulatory molecules. Since previous studies have suggested that EphB2 could be a co-stimulatory molecule for CD4+ T cell activation we asked whether ligation of EphB2 receptor expressed on the DC cell surface was modulated by ligation with Ephrin B ligands that are known to be expressed on the CD4+ T cell surface. Plate bound EphrinB1 (Fig 4A) or EphrinB2 ligand (Fig 4D) upregulated transcription of *EphB2* at 3 hours post-stimulation but for ephrin B1 stimulation this only occurred in the presence of IFN-γ. The efficacy of the stimulation was assessed by global phosphorylation of tyrosine kinases in the DCs, and EphrinB2 (Fig 4E and 4F), the main ligand for EphB2, was found to induce more sustained phosphorylation than EphrinB1 (Fig 4B and 4C). This indicates that ligation of EphB2 upregulates transcription of EphB2 in a positive feedback loop, and not all Ephrin B ligand family members may equivalent effects on DCs activation.

**Fig 5:**
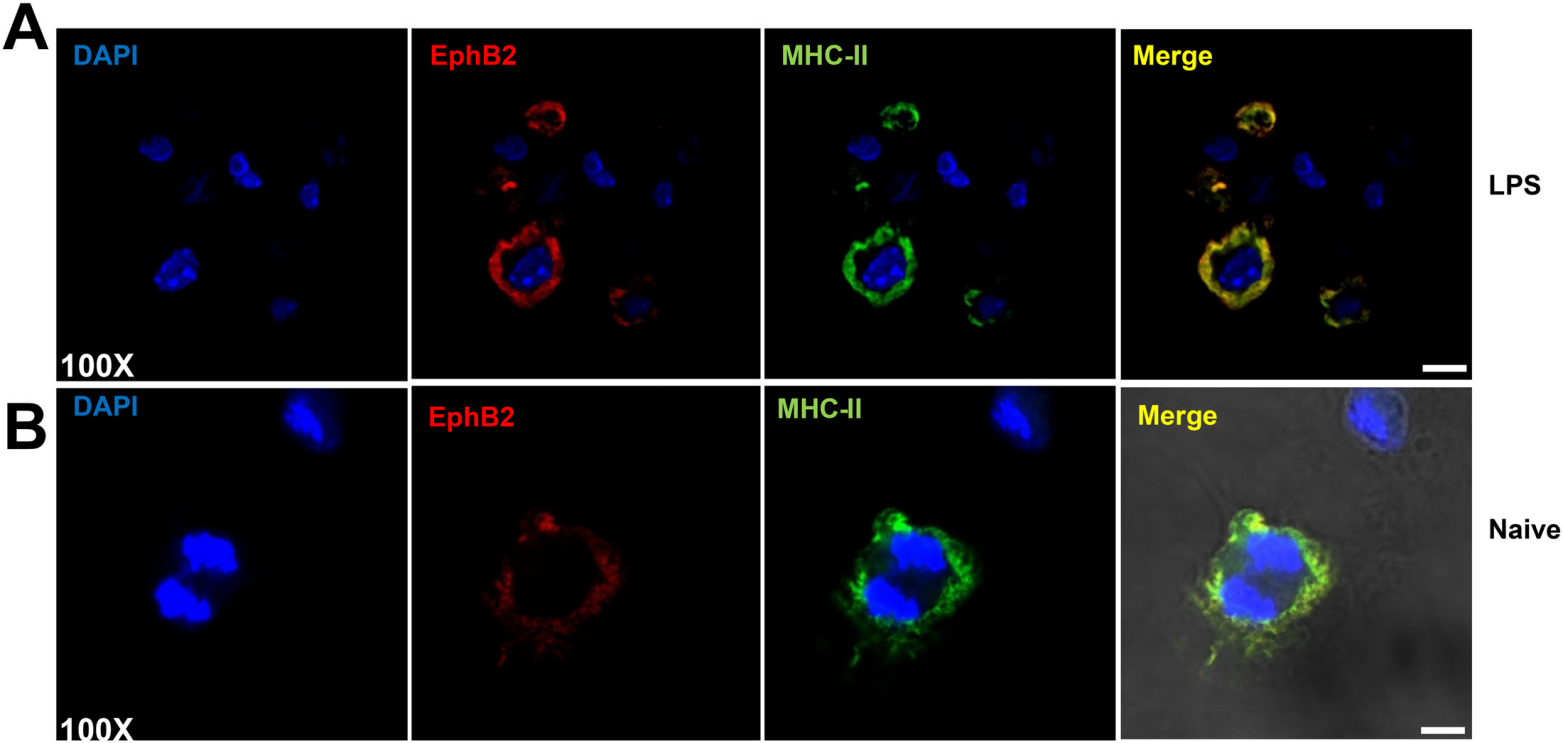
EphB2 co-localizes with MHC-II on BMDCs. Two examples are shown: (A) Representative BMDCs stimulated with 1μg/ml lipopolysaccharide (LPS) and 20ng/ml recombinant mouse interferon (IFN)-γ for 22 hours; (B) shows unstimulated cells. EphB2 is shown in red (Northernlights557), MHC-II is shown in green (FITC) and DAPI is shown to demarcate the nuclei of the cells. Magnification 100x; Scale bar, 20μm.

When T cells interact with DCs, peptide-loaded MHC-II molecules co-localize with costimulatory molecules in lipid rafts [28]. For EphB2 to act as a co-stimulatory molecule for CD4+T cells, it might be expected that this molecule would co-localize with MHC-II on DCs. The expression of MHC-II and EphB2 was analyzed on LPS-stimulated DCs by immunohistochemistry and both molecules were found to co-localize (Fig 5) providing evidence that EphB2 potentially works in concert with MHC-II in CD4+ T cell activation.

### EphB2 deficiency does not affect T cell activation *in vitro*

**Fig 6:**
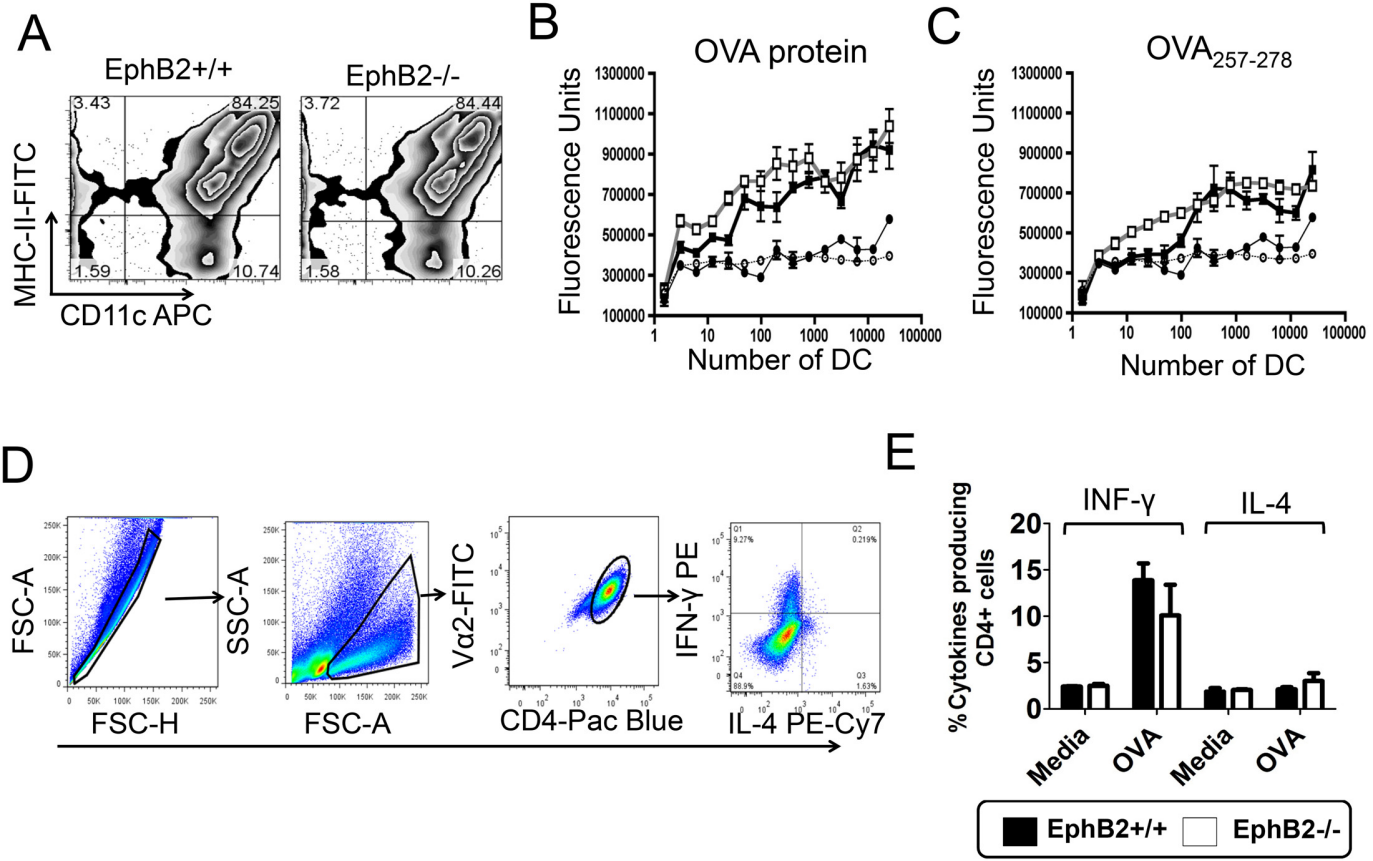
EphB2 is not required for development of Th1 cells in culture. BMDCs were matured from mouse bone marrow (A) and incubated with the MF2.2D9 T cell hybridoma and chicken ovalbumin (OVA) (B) or OVA_257-278_ peptide (C) for 18 hours. The amount of IL-2 secreted by the MF2.2D9 T cell hybridoma was measured using HT-2 cells and the cell number enumerated using an ATP-luminescence assay. The graphs represent the mean value ±SD of 3 replicate wells for each dilution of EphB2-/- BMDCs (gray lines) or littermate control BMDCs (black lines). Circles represent no addition of a stimulus and stimulated BMDCs are represented by squares. (D) BMDCs derived from bone marrow of EphB2-/- or EphB2+/+ mice were incubated with splenic CD4+ T cells purified from naïve OT-II T cell receptor transgenic mice (n = 5) and OVA protein for 5 days. The development and expansion of Th1 and Th2 cells were measured by intracellular staining of interferon (IFN)-γ and interleukin (IL)-4 respectively (E). All graphs are representative of at least 2 experiments, with the exception of (E) which is the median value of pooled data ±SD from 3 separate preparations of BMDCs.

Several co-stimulatory molecules have been described, and some studies have suggested that the combination of molecules providing co-stimulation to stimulate CD4+ T cells may influence the phenotype of T helper cells that develop. We tested whether EphB2 influenced the activation of CD4+T cells and the development of a Th1 phenotype as defined by their production of IFN-γ. CD11c+ MHC-II+ BMDCs were matured from bone marrow of EphB2-/- or EphB2+/+ littermate control mice (Fig 6A). To investigate whether EphB2 influenced the ability of BMDCs to process and present antigen to CD4+T cells BMDCs were cultured with OVA protein or the OVA_257-278_ peptide and the MF2.2D9 T cell hybridoma engineered to express T cell receptors specific for OVA_257-278_ [29]. Upon recognition of OVA_257-278_-loaded MHC-II on BMDCs, the MF2.2D9 T cell hybridoma will secrete IL-2. As measured by a cell proliferation bioassay, there was no significant difference in the secretion of IL-2 by MF2.2D9 cells incubated with EphB2-/- BMDCs when compared with EphB2+/+ BMDCs in the presence of OVA protein (Fig 6B) or OVA_257-278_ peptide (Fig 6C). This indicates that EphB2 deficiency does not affect the processing and presentation of peptide to CD4+T cells. To assess whether EphB2 affected the skew of cytokines produced by CD4+T cells upon activation, EphB2-/- or EphB2+/+ BMDCs were cultured for 5 days with OVA protein and OT-II T cells purified from the spleens of naïve OT-II T cell receptor transgenic mice that have CD4+T cells expressing T cell receptors specific for the OVA_323-339_ peptide of OVA [21]. By intracellular staining and flow cytometry, we were able to detect OT-II CD4+T cells expressing IFN-γ (Fig 6D) but not IL-4 (Fig 6E) in this assay. However, there was no statistically significant difference between the percentages of IFN-γ+CD4+ Th1 cells activated by BMDCs with an EphB2 deficiency compared with intact BMDCs (Fig 6E). Collectively these data indicate that EphB2 expression on DCs is not required for the development of IFN-γ producing Th1 cells.

### Redundancy in DC-expressed EphB receptor family members may explain the negligible role of EphB2 in CD4+ T cell activation

**Fig 7:**
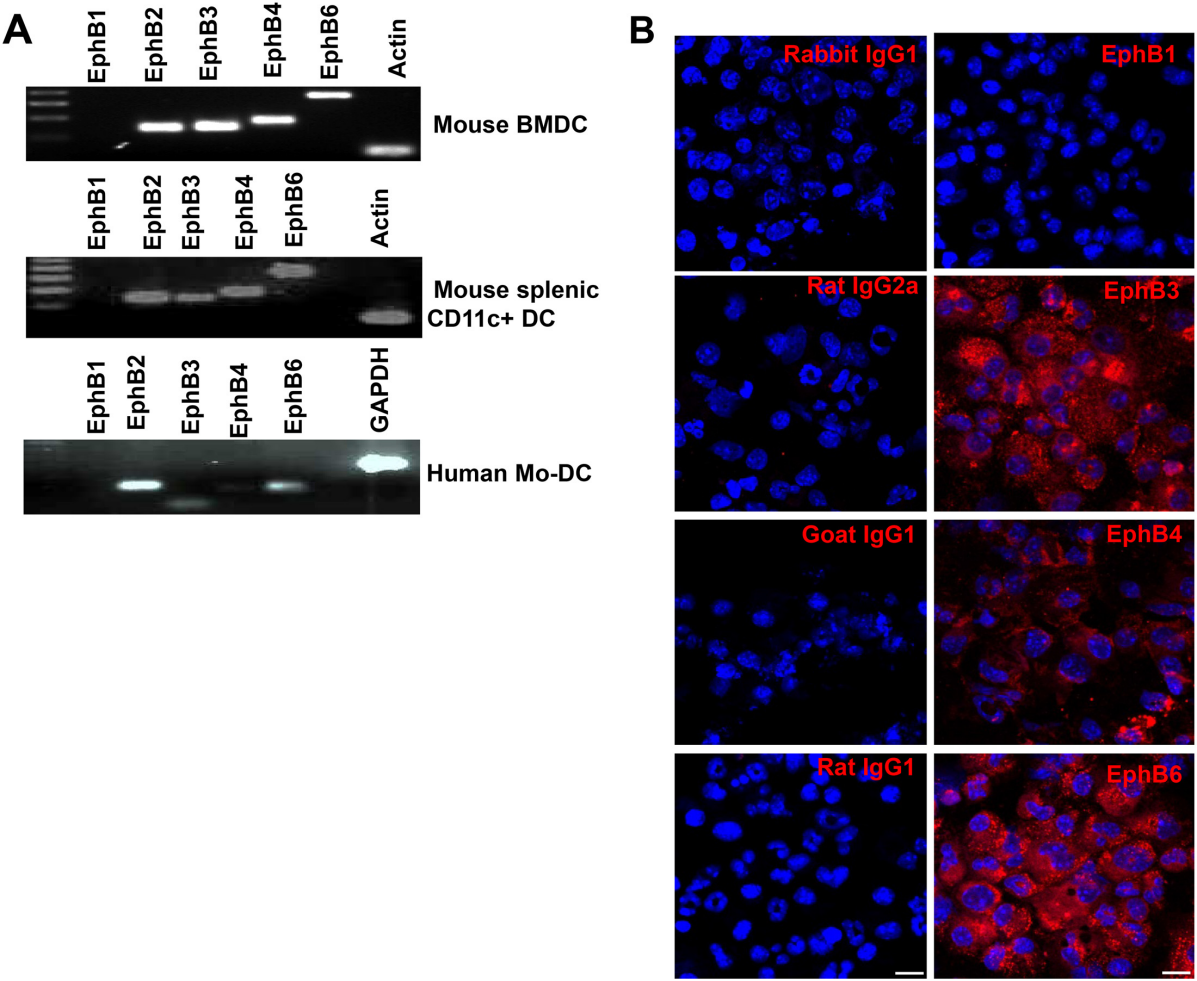
DCs express Eph receptors other than EphB2. RNA was extracted from naïve bone marrow-derived DCs (A), splenic DCs (B) and human monocyte-derived DCs (C) and PCR performed using primers specific for mouse and human EphB receptors. The protein expression of the EphB receptor family was detected using immunohistochemistry on naïve mouse BMDCs (D) and the expression compared to that detected using isotype controls. Magnification 40x; Scale bar, 10μm.

The EphB receptor family is promiscuous with respect to binding to Ephrin B ligands [30]. In a given cell type, the absence of one member of this family of receptor kinases could be complemented/compensated by the others conferring functional redundancy [31]. By looking at the breadth of expression of EphB receptors on naïve BMDCs we show that mouse BMDCs transcribe RNA for *EphB3*, *EphB4* and *EphB6* in addition to *EphB2* (Fig 7A) and confirmed that this transcription pattern was mirrored by the expression of these molecules using immunohistochemistry of BMDCs (Fig 7B). We found a similar pattern of transcription in mouse splenic CD11c+ DC (Fig 7A). EphB1 was not transcribed or expressed on mouse splenic CD11c+ or bone marrow-derived DCs (Fig 7B). To determine whether this pattern is mirrored in human DCs, we tested transcription of EphB receptor family members in human monocyte-derived DC, and found that *EphB3* and *EphB6* are transcribed in addition to *EphB2* confirming that both mouse and human DCs appear to express multiple members of the EphB receptor family (Fig 7B).

Since DCs can express several members of the EphB receptor family, we determined whether transcriptions of EphB receptors other than EphB2 were modulated by TLR stimulation. We undertook qPCR of BMDCs stimulated with LPS or CPG1668 and observed that, in addition to EphB2, EphB3 was upregulated by both stimuli (S2 Fig), demonstrating that EphB2 and EphB3 are co-modulated by TLR stimulation of BMDCs in mice. This lends weight to the hypothesis that Eph receptor redundancy may explain the lack of an effect of EphB2 deficiency on DC activation of CD4+T cells. EphB4 and EphB6 mRNA and proteins were not modulated in these assays (data not shown).

## Discussion

The role of the Eph receptor and ephrin ligand family of molecules in the induction of immune responses has not been adequately determined. The rationale that these molecules may be involved in immune responses is driven by the expression of these molecules in multiple immune cell types including DCs [3, 17, 18], T cells [9, 32] and monocytes [20]. They are also expressed on cells that are known to interact with the immune system including endothelial cells [33] and platelets [34]. Despite this, very little is known about their exact function and how they are regulated in response to the many signals that immune cells receive during an immune response.

Mature T cells from mice express EphB receptors [11, 35, 36] as well as Ephrin B ligands [10, 11]. Given that the function of Ephrin B ligands has been investigated on T cells in several reports [12, 15, 32], we set out to examine the expression of EphB2 receptor on DCs, the main antigen presenting cells that interact with T cells at the initiation of an immune response. Our data show that mouse and human-derived myeloid DC express EphB receptors (Figs 1 and 7). However, contrary to other reports demonstrating the expression of EphB1 receptor on Langerhans cells and interstitial DCs [3, 17], mouse BMDCs and CD11c^hi^ splenic DC transcribed RNA for all EphB receptors apart from EphB1 (Fig 7A and 7B), a pattern mirrored by the transcription profile in human Mo-DC (Fig 7A).

We reasoned that once DCs become activated in response to antigenic stimuli they will up-regulate various molecules at their surface, including the EphB2 receptor, and this will facilitate their binding to, and interaction with, EphrinB-expressing T cells. The immune response will normally be initiated by activation of DCs upon ligation of pattern recognition receptors (PRRs) such as TLRs by molecules containing pathogen-associated molecular patterns (PAMPs) [37] at the site of an infection. We have shown that EphB2 is up-regulated by TLR ligation on BMDCs (Fig 2) and also in a feedback loop upon ligation with EphrinB ligands (Fig 4). EphB receptors expressed on T cells have been shown to co-localize with the T cell receptor [10, 11, 13] suggesting that this family of molecules are intimately interlinked with the TCR-MHC interface in the interaction between T cells and antigen presenting cells. However given that DC-expressed ephrin B ligands are not modulated by TLR stimulation (S1 Fig) we focused on the role of T cell-expressed ephrin B ligands in T cell activation upon ligation with DC-expressed EphB receptors.

Migration of activated DCs to the draining lymph nodes results in interaction with T cells in the T cell zone. In addition to co-stimulation, and secretion of cytokines, the presentation of pathogen-derived peptides in the context of MHC molecules on the surface of the DC to T cell receptors drives the activation of T cells and initiates cell-mediated immune responses. Here we observed co-localization between EphB2 and MHC-II on the surface of activated BMDCs (Fig 5) suggesting a role for EphB2 in CD4+T cell activation. Given that macrophage expressed EphB2 supports adhesion to EphrinB2-expressing endothelial cells [33], we hypothesized that DCs that do not express EphB2 may have an impaired ability to adhere to ephrinB expressing CD4+T cells in turn altering T cell activation. However we did not find that EphB2 expression on DC was necessary for induction of CD4+T cell activation (Fig 6E).

The proliferation and differentiation of subsets of CD4+T cells secreting particular cytokines is required to orchestrate effector functions appropriate to killing of different invading pathogens [28, 37]. In this study we examined how EphB receptors expressed on DCs may influence the development of IFN-γ-secreting Th1 cells and IL-4-secreting Th2 cells, focusing on the role of EphB2 using BMDCs from EphB2 deficient [22] or intact mice as antigen presenting cells. We did not see any significant level of IL-4 producing OT-II T cells in this assay but equivalent numbers of IFN-γ producing OT-II cells expanded when stimulated with EphB2-/- or EphB2+/+ DCs (Fig 6E). This is consistent with the equivalent levels of IL-12p70 expressed by BMDCs matured from EphB2-/- or EphB2+/+ animals upon TLR ligation (Fig 3). We hypothesize that this result may have arisen due to redundancy between the members of the EphB receptor family that are expressed on DCs (Fig 7) and also modulated in response to TLR ligation (Fig 2 and S2 Fig).

Although we were unable to demonstrate any effect of ephrin B ligand stimulation by DC-expressed EphB2 on Th1 development the expression of EphB receptors on T cells raises the possibility that T cells use their EphrinB ligands to ligate with EphB receptors on other T cells. Indeed this phenomenon has been described for other molecules known to function as co-stimulatory molecules, such as the B7 family members CD80 and CD86 and CD28 [38]. Ligation of EphB receptors on the T cell surface induces signaling pathways in T cells that result in proliferation and activation of T cells, albeit to a lesser extent than with CD28 stimulation [10, 11]. EphB6 has been shown to generally be required for optimal production of IL-2, IFN-γ and IL-4 production from T cells in response to α-CD3 / α-CD28 stimulation even though it does not contain an active intracellular kinase domain [35, 39]. Interestingly the ligation of EphB receptors on the T cell surface at the time of stimulation leads to the development of Th1, but not Th2 cells [10]. Although there may be differing role of EphB and ephrin ligation on the T cell surface with respect to the development of Th1 cells, there has been no reported role for potential co-stimulation of T cells by EphB / ephrinB molecules specifically leading to development of Th2 cells. Our data suggest that the B family of Eph / ephrins are not involved in Th2 development.

In summary, our data show for the first time that TLR ligation can modulate the expression of EphB receptors on DCs. Although EphB2 co-localizes with MHC-II suggesting a role for T cell activation, we also show that DC-expressed EphB2 is not a required molecule for CD4+T cell activation and Th1 development. We hypothesize a redundancy in the roles that DC-expressed EphB receptors play in CD4+T cell activation and our data suggest additional studies should be carried out to elucidate the other functions that EphB receptors might play in DCs biology.

## Supporting Information

S1 Table Primers used for qPCR. (DOC)

S1 Fig Transcription of *EphrinB* ligands is mostly downregulated by ligation with Toll-like receptor ligands. (A) BMDCs were incubated with a Toll-like receptor (TLR)4 agonist (lipopolysaccharide (LPS) 1μg/ml) and (B) a TLR9 agonist (CpG1668 1μM) +/- recombinant mouse interferon (IFN)-γ 20ng/ml and *EphrinB1*, *EphrinB2 and EphrinB3* mRNA quantified by qPCR at different time points post-stimulation.
(TIF)

S2 Fig Transcription of *EphB3* is modulated by ligation with Toll-like receptor ligands. (A) BMDCs were incubated with a Toll-like receptor (TLR)4 agonist (lipopolysaccharide (LPS) 1μg/ml) and (B) a TLR9 agonist (CpG1668 1μM) +/- recombinant mouse interferon (IFN)-γ 20ng/ml and *EphB3* mRNA quantified by qPCR at different time point post-stimulation. (C) The change in EphB3 protein expression at 22 hours post-incubation with LPS, CpG1668 and recombinant mouse IFN-γ is shown and the mean fluorescence quantified for different conditions. All graphs represent the median value of pooled data across 3 independent dendritic cell preparations ±SD and data analyzed using One-way ANOVA– Kruskal Wallis test and Dunn’s multiple comparisons post-test. *P<0.05. MFI = Mean Fluorescence Intensity.
(TIF)
