# Supplementary material for: Expression of the Receptor Tyrosine Kinase EphB2 on Dendritic Cells Is Modulated by Toll-Like Receptor Ligation but Is Not Required for T Cell Activation
Source: PLoS One. 2015 Sep 25;10(9):e0138835. doi: 10.1371/journal.pone.0138835 (PMC4583388; doi:10.1371/journal.pone.0138835)
Supplement: S1 Table — (DOC) [file pone.0138835.s001.doc]

Table S1: primers used for qPCR

| **Gene** | **Forward primer** | **Reverse primer** |
| --- | --- | --- |
| ephrin B1(mouse) | TCGCAAGCATACACAGCAGCGG | ATGATGATGTCGCTGGGCTCGG |
| ephrin B2(mouse) | CAGAAGAACCCTGCTTGCCTGG | AGCAAGCAGCCTTGACCTGC |
| ephrin B3(mouse) | AGACTTTGGGGGAGTTGGTGCC | CAGCCCCGCAAAACCTAACAGC |
| EphB1 (mouse) | TTACAGCACAGGCCGAGGGGAGTTCG | AACTGGCCCATGATGCTCGCC |
| EphB2 (mouse) | ACGCCACGGCCATAAAAAGCCC | TTGCCACTGTAGCGCCCATAGC |
| EphB3 (mouse) | ATTGGGCATCAAGCCACCCAGC | TGCTCTGTAACCGAGGTGTCGC |
| EphB4 (mouse) | TTGAGCCCTGGGTGGCAATCCG | AGGCACCTCACGGTCAGTGG |
| EphB6 (mouse) | ACTCTAAGCTGCGAGCAGACGC | GCCAGGCTTGCCTTCTTGTCTGG |
| Β-actin (mouse) | TGTGCTGTCCCTGTATGCCTCTGG | GGGAGAGCATAGCCCTCGTAGATGG |
| Ubiquitin (mouse) | TGGCTATTAATTATTCGGTCTGCAT | GCAAGTGGCTAGAGTGCAGAGTAA |
| EphB1 (human) | AAAGGATACCGAGAAGCCACCCGC | AGATAATCCAGGGCCATCGCCG |
| EphB2 (human) | ACGCACCGTGGCAGGCTACG | GGCGATGACAACCACAGCAATGAGG |
| EphB3 (human) | ACCGCCAGATTACTGTGCATCCCG | GAGCTGGGTGCCTGAGGACTTGGG |
| EphB4 (human) | CCGCGCGGAGTATCGGCG | CTTCCAAAGCTGCGGCCAACG |
| EphB6 (human) | CCGCTGCCAGCCTGGATACC | CTCCAGGCAGGGGCAAACGG |
| GAPDH(human) | TCAGTTGTAGGCAAGCTGCGACGT | AAGCCAGAGGCTGGTACCTAGAAC |
| CD80 (mouse) | TAACTGAGTCTGGAAACCCATCTG | GAAGCGAGGCTTTGGGAAAC |
| CD86 (mouse) | GAAACTTGATAGTGTGAATGCCAAGTA | GAAGTCGTAGAGTCCAGTTGTTCCT |
| IL-12p40 (mouse) | GTGGGTGTCCAGGCACATCAGACC | CACATCCCACTCCCACGCTGC |
