# Supplementary figures and images for: Expression of the Receptor Tyrosine Kinase EphB2 on Dendritic Cells Is Modulated by Toll-Like Receptor Ligation but Is Not Required for T Cell Activation
Source: PLoS One. 2015 Sep 25;10(9):e0138835. doi: 10.1371/journal.pone.0138835 (PMC4583388; doi:10.1371/journal.pone.0138835)

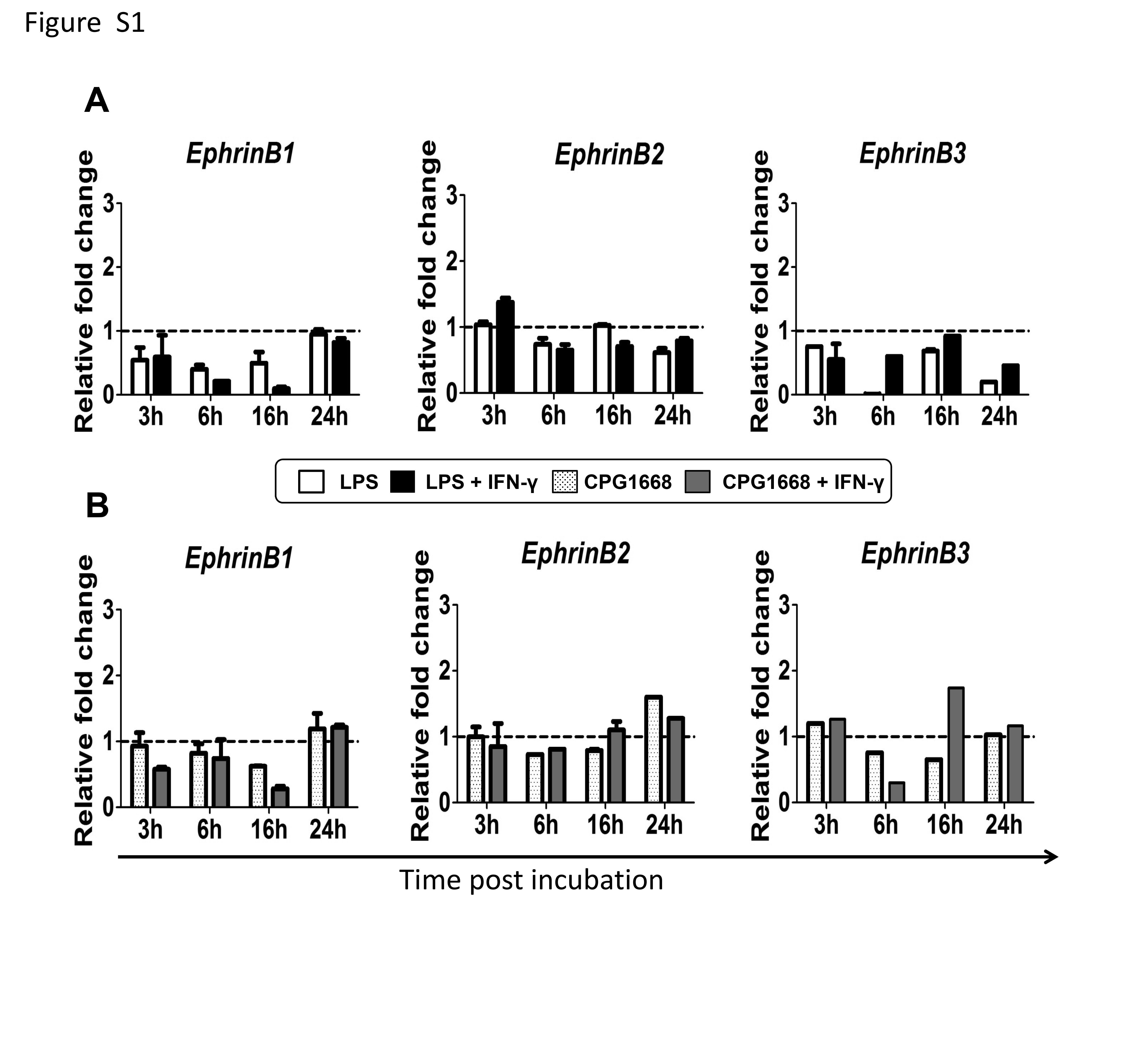

Supplement: S1 Fig — (A) BMDCs were incubated with a Toll-like receptor (TLR)4 agonist (lipopolysaccharide (LPS) 1μg/ml) and (B) a TLR9 agonist (CpG1668 1μM) +/- recombinant mouse interferon (IFN)-γ 20ng/ml and EphrinB1, EphrinB2 and EphrinB3 mRNA quantified by qPCR at different time points post-stimulation. (TIF) [file pone.0138835.s002.tif]

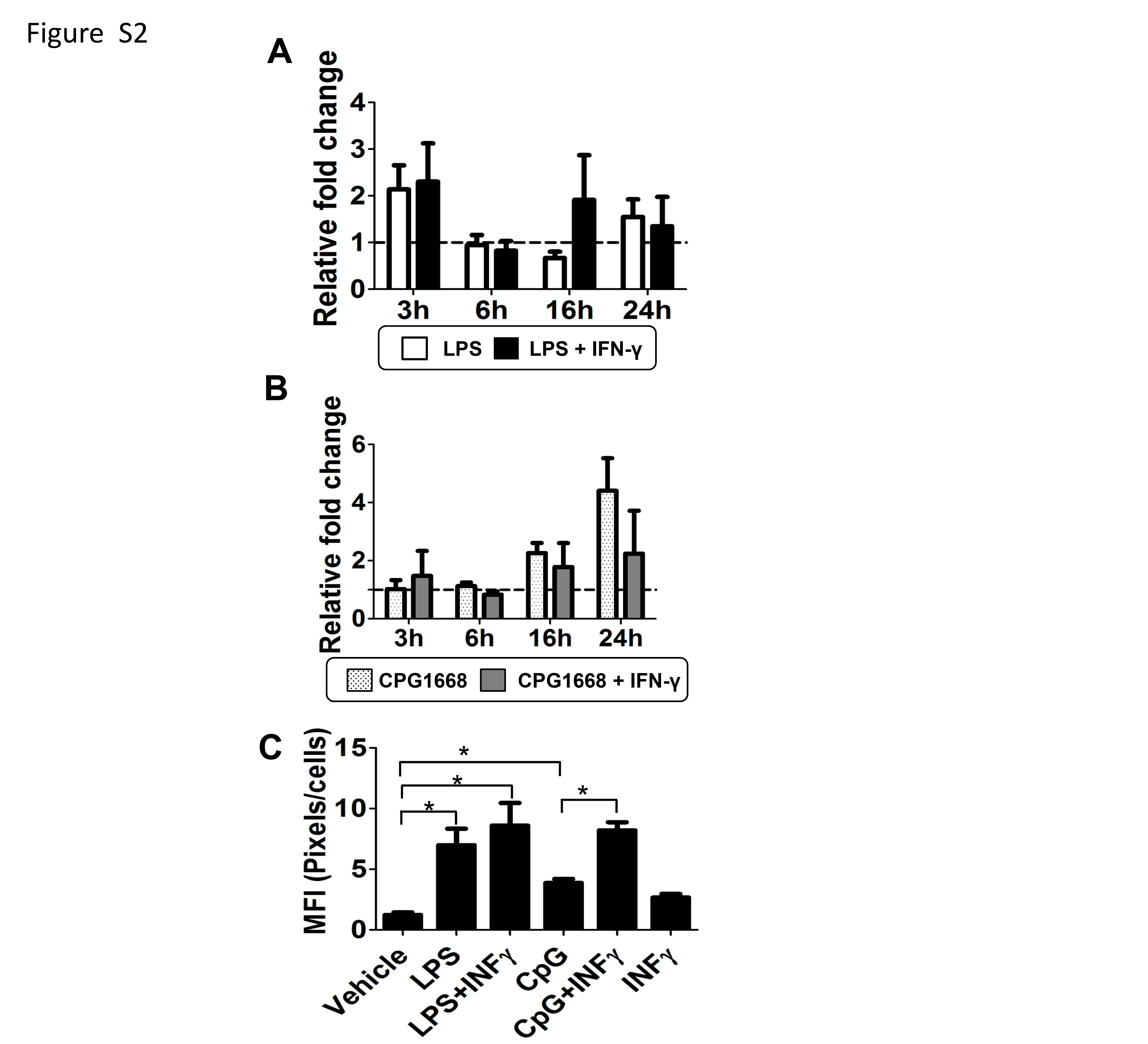

Supplement: S2 Fig — (A) BMDCs were incubated with a Toll-like receptor (TLR)4 agonist (lipopolysaccharide (LPS) 1μg/ml) and (B) a TLR9 agonist (CpG1668 1μM) +/- recombinant mouse interferon (IFN)-γ 20ng/ml and EphB3 mRNA quantified by qPCR at different time point post-stimulation. (C) The change in EphB3 protein expression at 22 hours post-incubation with LPS, CpG1668 and recombinant mouse IFN-γ is shown and the mean fluorescence quantified for different conditions. All graphs represent the median value of pooled data across 3 independent dendritic cell preparations ±SD and data analyzed using One-way ANOVA– Kruskal Wallis test and Dunn’s multiple comparisons post-test. *P<0.05. MFI = Mean Fluorescence Intensity. (TIF) [file pone.0138835.s003.tif]
